# Urinary Excretion of Fatty Acid-Binding Protein 4 is Associated with Albuminuria and Renal Dysfunction

**DOI:** 10.1371/journal.pone.0115429

**Published:** 2014-12-15

**Authors:** Yusuke Okazaki, Masato Furuhashi, Marenao Tanaka, Tomohiro Mita, Takahiro Fuseya, Shutaro Ishimura, Yuki Watanabe, Kyoko Hoshina, Hiroshi Akasaka, Hirofumi Ohnishi, Hideaki Yoshida, Shigeyuki Saitoh, Kazuaki Shimamoto, Tetsuji Miura

**Affiliations:** 1 Department of Cardiovascular, Renal and Metabolic Medicine, Sapporo Medical University School of Medicine, Sapporo, Japan; 2 Department of Public Health, Sapporo Medical University School of Medicine, Sapporo, Japan; 3 Department of Nursing, Division of Medical and Behavioral Subjects, Sapporo Medical University School of Health Sciences, Sapporo, Japan; 4 Sapporo Medical University, Sapporo, Japan; Universtiy of Maryland School of Medicine, United States of America

## Abstract

**Background:**

Fatty acid-binding protein 4 (FABP4/A-FABP/aP2) is expressed in not only adipocytes and macrophages but also peritubular capillaries in the normal kidney. We recently demonstrated that ectopic expression of FABP4, but not FABP1 known as liver FABP (L-FABP), in the glomerulus is associated with progression of proteinuria and renal dysfunction. However, urinary excretion of FABP4 has not been investigated.

**Methods:**

Subjects who participated in the Tanno-Sobetsu Study, a study with a population-based cohort design, in 2011 (n = 392, male/female: 166/226) were enrolled. Urinary FABP4 (U-FABP4) and urinary albumin-to-creatinine ratio (UACR) were measured. Change in estimated glomerular filtration rate (eGFR) was followed up one year later.

**Results:**

In 93 (23.7%) of the 392 subjects, U-FABP4 level was below the sensitivity of the assay. Subjects with undetectable U-FABP4 were younger and had lower UACR and higher eGFR levels than subjects with measurable U-FABP4. U-FABP4 level was positively correlated with age, systolic blood pressure and levels of serum FABP4 (S-FABP4), triglycerides, hemoglobin A1c (HbA1c), urinary FABP1 (U-FABP1) and UACR (r = 0.360, p<0.001). Age, S-FABP4, U-FABP1 and UACR were independent predictors of U-FABP4. On the other hand, systolic blood pressure, HbA1c and U-FABP4 were independently correlated with UACR. Reduction in eGFR after one year was significantly larger in a group with the highest tertile of baseline U-FABP4 than a group with the lowest tertile.

**Conclusions:**

Urinary FABP4 level is independently correlated with level of albuminuria and possibly predicts yearly decline of eGFR. U-FABP4 would be a novel biomarker of glomerular damage.

## Introduction

Fatty acid-binding proteins (FABPs) are proteins of about 14–15 kDa in size that can reversibly bind to hydrophobic ligands, such as long chain fatty acids, with high affinity and coordinate lipid responses in cells [1], [2]. FABPs have been proposed to facilitate the transport of lipids to specific compartments in the cell. Among FABPs, FABP1, known as liver FABP (L-FABP), is expressed in proximal tubular epithelial cells in the kidney [3]. It has been reported that urinary FABP1 reflects damage of proximal tubular epithelial cells [4], [5] and predicts progression of renal dysfunction [6], [7].

FABP4, also known as adipocyte FABP (A-FABP) or aP2, is expressed in both adipocytes and macrophages and plays important roles in the development of insulin resistance and atherosclerosis [8]–[11]. It has been shown that a small-molecule specific FABP4 inhibitor would be a novel strategy to prevent and treat type 2 diabetes mellitus and atherosclerosis [12]. Recent studies also showed that FABP4 is one of the novel adipocyte-derived bioactive molecules referred to as adipokines [13] and that elevated circulating FABP4 level is associated with obesity, insulin resistance, hypertension, cardiac dysfunction and atherosclerosis [14]–[20].

Other than adipocytes and macrophages, it has been reported that FABP4 is expressed in endothelial cells of capillaries and small veins, but not arteries, in several mouse and human tissues including the heart and kidney [21], [22]. Recently, we demonstrated that ectopic FABP4 expression in the glomerulus is associated with progression of proteinuria and renal dysfunction [23]. However, significance of urinary excretion of FABP4 has not been elucidated. We here investigated the association between urinary excretion of FABP4 and renal function in the Tanno-Sobetsu study, a prospective cohort study.

## Methods

### Study subjects

The Tanno-Sobetsu Study is a study with a population-based cohort design recruiting residents of two rural towns, Tanno and Sobetsu, in Hokkaido and includes voluntarily annual health examination and follow-up survey. Of subjects who participated in the Tanno-Sobetsu Study, a total of 392 subjects (male/female: 166/226) in Sobetsu Town were enrolled for the present analyses in 2011. This study conformed to the principles outlined in the Declaration of Helsinki and was performed with the approval of the ethical committee of Sapporo Medical University. Written informed consent was received from all of the subjects.

### Measurements

Medical check-ups were performed between 06:00 h and 09:00 h after an overnight fast. After measuring anthropometric parameters, blood pressure was measured twice consecutively on the upper arm using an automated sphygmomanometer (HEM-907, Omron Co., Kyoto, Japan) with subjects in a seated resting position, and average blood pressure was used for analysis. Body mass index (BMI) was calculated as body weight (in kilograms) divided by the square of body height (in meters). Peripheral venous blood and urine samples were obtained from study subjects after physical examination. The serum, plasma and urine samples were analyzed immediately or stored at −80°C until biochemical analyses.

Concentrations of FABP4 in serum (S-FABP4) and urine (U-FABP4) and FABP1 in urine (U-FABP1) were measured using commercially available enzyme-linked immunosorbent assay kits of FABP4 (Biovendor R&D, Modrice, Czech Republic) and FABP1 (CIMIC Co., Tokyo, Japan). The accuracy, precision and reproducibility of the kits have been described previously [14], [24]. Levels of U-FABP4 and U-FABP1 were normalized by urine creatinine level (µg/gCr).

Fasting plasma insulin was measured by a radioimmunoassay method. Creatinine (Cr) and lipid profiles, including total cholesterol, high-density lipoprotein (HDL) cholesterol and triglycerides, were determined by enzymatic methods. Low-density lipoprotein (LDL) cholesterol level was calculated by the Friedewald equation. Hemoglobin A1c (HbA1c) was determined by a latex coagulation method and was expressed in national glycohemoglobin standardization program (NGSP) scale. High-sensitivity C-reactive protein (hsCRP) was measured by a nephelometry method. HOMA-R, an indicator of insulin resistance, was calculated by the previously reported formula: insulin (µU/ml) × glucose (mg/dl)/405. Urinary albumin-to-creatinine ratio (UACR; mg/gCr) was used as a marker of microalbuminuria. Estimated glomerular filtration rate (eGFR) was calculated by an equation for Japanese [25]: eGFR (mL/min/1.73 m^2^)  = 194×Cr^(−1.094)^×age^(−0.287)^ ×0.739 (if female). For assessing yearly decline of eGFR, change in eGFR was calculated: eGFR in 2012 – eGFR in 2011.

### Statistical analysis

Numeric variables are expressed as means ±SD or medians (interquartile ranges). The distribution of each parameter was tested for its normality using the Shapiro-Wilk W test, and non-normally distributed parameters were logarithmically transformed. Differences in parameters between two groups were tested by the unpaired t test. The correlation between two variables was evaluated using Pearson's correlation coefficient. One-way analysis of variance and Tukey-Kramer *post hoc* test were used for detecting significant differences in data among three groups. Multiple linear regression analysis was performed to identify independent determinants of U-FABP4, UACR and change in eGFR. A p value of less than 0.05 was considered statistically significant.

## Results

### Characteristics of the study subjects

Basal characteristics of the study subjects are shown in Table 1. Female subjects had significantly smaller waist circumference, lower levels of glucose and Cr and higher levels of total cholesterol, HDL cholesterol, LDL cholesterol and S-FABP4 than did male subjects. The underlying diseases were hypertension (n = 206, 52.6%), diabetes mellitus (n = 49, 12.5%), dyslipidemia (n = 92, 23.5%), ischemic heart disease (n = 19, 4.8%) and stroke (n = 8, 2.0%). Of the 392 subjects, 123 (31.4%) were not on any medication.

**Table 1:** Characteristics of the studied 392 subjects.

	Whole	Male	Female
n	392	166	226
Age (years)	74±7	74±7	74±7
Body mass index (kg/m^2^)	23.6±3.6	24.0±3.4	23.3±3.7
Waist circumference (cm)	86±10	87±9	84±10**
Systolic blood pressure (mmHg)	145±22	144±20	146±23
Diastolic blood pressure (mmHg)	78±12	78±13	78±12
Biochemical data			
Total cholesterol (mg/dl)	200±32	190±31	208±20**
HDL cholesterol (mg/dl)	66±17	62±16	69±30**
LDL cholesterol (mg/dl)	122±28	115±29	126±26**
Triglycerides (mg/dl) ^b^	89 (66–120)	86 (66–120)	92 (66–121)
Glucose (mg/dl)	101±24	105±26	99±23*
HbA1c (%)	5.7±0.6	5.7±0.7	5.6±0.6
Insulin (µU/ml) ^b^	4.9 (3.3–7.3)	4.9 (3.3–7.3)	4.9 (3.4–7.2)
HOMA-R ^b^	1.2 (0.8–1.9)	1.3 (0.8–1.9)	1.1 (0.8–1.9)
Blood urea nitrogen (mg/dl)	17±4	17±5	16±4
Creatinine (mg/dl)	0.77±0.17	0.88±0.15	0.69±0.14**
Estimated GFR (ml/min/1.73 m^2^)	66±12	67±11	65±13
hsCRP (mg/dl) ^b^	0.04 (0.02–0.10)	0.05 (0.03–0.10)	0.04 (0.02–0.10)
S-FABP4 (ng/ml) ^b^	13.6 (9.3–18.8)	9.76 (6.6–14.6)	15.5 (12.0–20.2)**
Urinary examination			
UACR (mg/gCr) ^b^	10.2 (5.5–21.8)	8.6 (4.6–20.0)	10.9 (6.5–22.4)
U-FABP1 (µg/gCr) ^b^	5.5 (3.7–8.3)	4.6 (3.1–7.4)	6.2 (4.4–9.2)
U-FABP4 (µg/gCr) ^b^	0.25 (0.10–0.71)	0.20 (0.07–0.56)	0.29 (0.11–0.74)
non-detection of U-FABP4 ^a^	93 (23.7)	50 (30.1)	43 (19.0)
Diagnosis ^a^			
Hypertension	206 (52.6)	93 (56.0)	113 (50.0)
Diabetes mellitus	49 (12.5)	27 (16.3)	22 (9.7)
Dyslipidemia	92 (23.5)	26 (15.7)	66 (29.2)*
Ischemic heart disease	19 (4.8)	11 (6.6)	8 (3.5)
Stroke	8 (2.0)	7 (4.2)	1 (0.4)*
Medication (−)	123 (31.4)	50 (30.1)	73 (32.3)

Variables are expressed as means ±SD, ^a^ number (%), or ^b^ medians (interquartile ranges).

FABP, fatty acid-binding protein; GFR, glomerular filtration rate; hsCRP, high-sensitivity C-reactive protein; UACR, urine albumin-to-creatinine ratio.

*P<0.05,

**P<0.01 vs. male.

### Association of urinary FABP4 level with clinical characteristics

In 93 (23.7%) of the 392 subjects (male/female: 50/43), U-FABP4 level was below the sensitivity of the assay (i.e., <0.1 ng/ml). In the other 299 subjects, U-FABP4 could be determined and its level normalized by urinary Cr ranged from 0.01 to 38.6 µg/gCr. As shown in Table 2, subjects with undetectable levels of U-FABP4 were younger and had lower levels of triglycerides, S-FABP4, UACR and U-FABP1 and higher level of eGFR than subjects with measurable U-FABP4.

**Table 2:** Characteristics of the subjects with and without detectable U-FABP4.

	Undetectable U-FABP4	Detectable U-FABP4
n (M/F)	93 (50/43)	299 (116/183)
Age (years)	71±6	75±7**
Body mass index (kg/m^2^)	23.2±3.2	23.7±3.7
Waist circumference (cm)	84±9	86±10
Systolic blood pressure (mmHg)	139±20	147±22
Diastolic blood pressure (mmHg)	78±12	78±12
Biochemical data		
Total cholesterol (mg/dl)	203±32	199±32
HDL cholesterol (mg/dl)	68±17	65±16
LDL cholesterol (mg/dl)	123±27	121±28
Triglycerides (mg/dl) ^b^	82 (64–114)	92 (68–128)*
Glucose (mg/dl)	100±18	102±26
HbA1c (%)	5.6±0.5	5.7±0.7
Insulin (µU/ml) ^b^	4.9 (3.5–7.1)	4.9 (3.3–7.5)
HOMA-R ^b^	1.1 (0.8–1.8)	1.2 (0.8–1.9)
Blood urea nitrogen (mg/dl)	17±4	17±4
Creatinine (mg/dl)	0.76±0.13	0.78±0.18
Estimated GFR (ml/min/1.73 m^2^)	68.6±8.7	64.9±13.1*
hsCRP (mg/dl) ^b^	0.05 (0.03–0.09)	0.04 (0.02–0.10)
S-FABP4 (ng/ml)^ b^	10.9 (7.2–16.1)	14.3 (10.0–19.7)**
Urinary examination		
UACR (mg/gCr) ^b^	5.9 (3.5–9.7)	12.7 (6.5–29.0)*
U-FABP1 (µg/gCr) ^b^	3.6 (2.8–4.8)	6.4 (4.4–9.2)**
Diagnosis ^a^		
Hypertension	36 (38.7)	170 (56.9)
Diabetes mellitus	8 (8.6)	41 (13.7)
Dyslipidemia	19 (20.4)	73 (24.4)
Ischemic heart disease	3 (3.2)	16 (5.4)
Stroke	2 (2.2)	6 (2.0)
Medication (−)	58 (62.4)	200 (66.9)

Variables are expressed as means ±SD, ^a^ number (%), or ^b^ medians (interquartile ranges).

FABP, fatty acid-binding protein; GFR, glomerular filtration rate; hsCRP, high-sensitivity C-reactive protein; UACR, urine albumin-to-creatinine ratio.

*P<0.05,

**P<0.01 vs. Undetectable FABP4.

**Figure 1:**
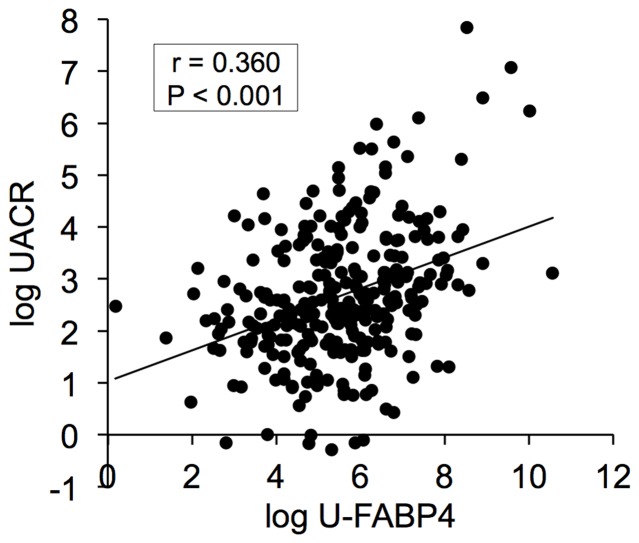
Correlation between urinary FABP4 and albuminuria. Logarithmically transformed urinary albumin-to-creatinine ratio (UACR) was plotted against logarithmically transformed urinary FABP4 (U-FABP4) for each subject with detectable U-FABP4 level (n = 299). There was a significant correlation between the two parameters (r = 0.360, p<0.001).

The level of U-FABP4 was significantly correlated with S-FABP4 (r = 0.280, p<0.001) (Table 3). In contrast to S-FABP4, which was significantly correlated with BMI (r = 0.480, p<0.001) and waist circumference (r = 0.402, p<0.001), U-FABP4 was not correlated with BMI or waist circumference. U-FABP4 was negatively correlated with insulin level (r = −0.115, p = 0.04) and positively correlated with age (r = 0.255, p<0.001), systolic blood pressure (r = 0.189, p = 0.001) and levels of triglycerides (r = 0.115, p = 0.04), HbA1c (r = 0.124, p = 0.03), UACR (r = 0.360, p<0.001; Fig. 1) and U-FABP1 (r = 0.534, p<0.001) (Table 3). Stepwise regression analysis using the correlated parameters revealed that age, S-FABP4, UACR and U-FABP1 were independent predictors of U-FABP4 (Table 3). A subsequent multiple regression analysis showed that age, S-FABP4, UACR and U-FABP1 were independently correlated with U-FABP4, explaining 40.2% of the variance in this measure (R^2^ = 0.402) (Table 3).

**Table 3:** Simple and multiple regression analyses for log U-FABP4 (n  =  299).

	For log U-FABP4			
	Simple regression		Stepwise regression	
	*r*	*P*	*t*	*P*
Age	0.255	<0.001	3.09	0.002
Gender (Male)	-	-	−0.18	0.854
Body mass index	0.078	0.181	-	-
Waist circumference	0.047	0.417	-	-
Systolic blood pressure	0.189	0.001	NS	
Diastolic blood pressure	0.029	0.619	-	-
Biochemical data				
Total cholesterol	−0.034	0.558	-	-
HDL cholesterol	−0.046	0.426	-	-
LDL cholesterol	−0.037	0.521	-	-
log Triglycerides	0.115	0.047	NS	
Glucose	0.050	0.386	-	-
HbA1c	0.124	0.032	NS	
log Insulin	−0.115	0.048	NS	
log HOMA-R	−0.089	0.124	-	-
Blood urea nitrogen	0.076	0.189	-	-
Creatinine	0.036	0.536	-	-
estimated GFR	−0.113	0.051	-	-
log hsCRP	0.071	0.232	-	-
log S-FABP4	0.280	<0.001	3.47	0.001
Urinary examination				
log UACR	0.360	<0.001	4.17	<0.001
log U-FABP1	0.534	<0.001	9.78	<0.001

FABP, fatty acid-binding protein; GFR, glomerular filtration rate; hsCRP, high-sensitivity C-reactive protein; UACR, urine albumin-to-creatinine ratio; NS, not selected.

UACR was negatively correlated with eGFR and positively correlated with age, systolic blood pressure, diastolic blood pressure and levels of triglycerides, glucose, HbA1c, blood urea nitrogen, S-FABP4 (r = 0.244, p<0.001), U-FABP1 (r = 0.254, p<0.001) and U-FABP4 (r = 0.360, p<0.001; Fig. 1) (Table 4). In stepwise regression analysis using the correlated parameters, age, systolic blood pressure, HbA1c and U-FABP4 were selected as independent predictors of UACR, and a subsequent multiple regression analysis showed that systolic blood pressure, HbA1c and U-FABP4 were independently correlated with UACR, explaining a total of 21.2% of the variance in this measure (R^2^ = 0.212) (Table 4). Furthermore, U-FABP4 was an independent predictor for UACR after adjustment of age, gender, systolic blood pressure, HbA1c, eGFR, S-FABP4 and U-FABP1.

**Table 4:** Simple and multiple regression analyses for log UACR (n = 392).

	For log UACR			
	Simple regression		Stepwise regression	
	*r*	*p*	*t*	*p*
Age	0.239	<0.001	1.87	0.062
Gender (Male)	-	-	−0.73	0.463
Body mass index	0.099	0.052	-	-
Waist circumference	0.078	0.126	-	-
Systolic blood pressure	0.291	<0.001	3.70	<0.001
Diastolic blood pressure	0.164	0.001	NS	
Biochemical data				
Total cholesterol	0.012	0.808	-	-
HDL cholesterol	−0.095	0.061	-	-
LDL cholesterol	0.032	0.523	-	-
log Triglycerides	0.107	0.035	NS	
Glucose	0.164	0.001	NS	
HbA1c	0.183	<0.001	3.51	0.001
log Insulin	0.027	0.599	-	-
log HOMA-R	0.069	0.171	-	-
Blood urea nitrogen	0.132	0.009	NS	
Creatinine	0.092	0.069	-	-
estimated GFR	−0.172	0.001	NS	
log hsCRP	0.033	0.523	-	-
log S-FABP4	0.244	<0.001	NS	
Urinary examination				
log UACR	-	-	-	-
log U-FABP1	0.254	<0.001	NS	
log U-FABP4	0.360	<0.001	4.78	<0.001

FABP, fatty acid-binding protein; GFR, glomerular filtration rate; hsCRP, high-sensitivity C-reactive protein; UACR, urine albumin-to-creatinine ratio; NS, not selected.

**Figure 2:**
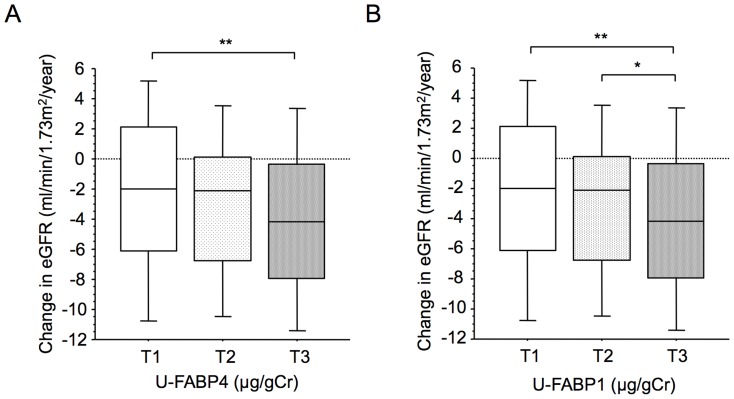
Change in eGFR in tertiles of urinary FABP4 and FABP1. **A, B.** Of the 392 recruited subjects, 325 subjects could be followed up in 2012, and change in eGFR from 2011 to 2012 (mL/min/1.73 m^2^/year) shown by box plots was compared among three groups divided by tertiles of urinary FABP4 (U-FABP4) (A) and urinary FABP1 (U-FABP1) (B). Tertile of U-FABP4 consists of low (T1<0.04 µg/gCr, n = 108), middle (0.04≤T2<0.30 µg/gCr, n = 108), and high (T3≥0.30 µg/gCr, n = 109) groups. Tertile of U-FABP1 consists of low (T1<4.27 µg/gCr, n = 108), middle (4.27≤T2<7.15 µg/gCr, n = 108), and high (T3≥7.15 µg/gCr, n = 109) groups. *P<0.05, **P<0.001.

Of the 392 study subjects, 325 subjects received follow up re-examination one year later (i.e., in 2012). The re-examined subjects were divided according to tertile of U-FABP4 or U-FABP1 at baseline: low (1^st^ tertile, T1), middle (2^nd^ tertile, T2) and high (3^rd^ tertile, T3) U-FABP groups. Of note, the subjects with undetectable U-FABP4 were assigned as low (T1) U-FABP4 group. As shown in Fig. 2A, reduction in eGFR was significantly larger in the high-U-FABP4 group than in the low-U-FABP4 group. Similarly, the high-U-FABP1 group showed larger reduction in eGFR than did the low- and middle-U-FABP1 groups (Fig. 2B).

Change in eGFR was negatively correlated with baseline UACR (r = −0.154, p = 0.005), eGFR (r = −0.323, p<0.001), U-FABP1 (r = −0.140, p = 0.029) and U-FABP4 (r = −0.140, p = 0.011) but was not correlated with S-FABP4 (r = 0.024, p = 0.655). Multiple regression analysis showed that U-FABP4 was an independent predictor for change in eGFR after adjustment of age, gender and baseline eGFR. However, U-FABP4 was not selected as an independent determinant of change in eGFR when UACR or U-FABP1 was additionally incorporated into the adjustment.

## Discussion

This is the first report regarding significance of urinary FABP4 level in a general population. In normal kidneys, FABP4 is expressed in endothelial cells of the tubulointerstitial peritubular capillary (PTC) and vein in both the cortex and medulla, but not in glomerular or arterial endothelial cells, under a normal physiological condition [21]. Interestingly, FABP4 was markedly and ectopically up-regulated in endothelial cells regenerating after endothelial balloon denudation in the pig coronary artery [26]. We recently demonstrated that FABP4 was remarkably expressed in the glomerulus in patients with strong inflammatory disorders of capillaries [23]. Furthermore, the level of FABP4 expression in the glomerulus was significantly higher in patients with endothelial proliferative lesions than in those without the lesions in IgA nephropathy. These findings indicate that ectopic expression of FABP4 in glomerular endothelial cells is associated with local inflammation in the glomerulus. In the previous study [23], we also demonstrated the association between increased glomerular FABP4 area, which was expressed in glomerular endothelial cells and macrophages, and decline in eGFR. Furthermore, the present study showed an association of high level of U-FABP4 with larger decline in eGFR (Fig. 2A). Taken together, U-FABP4 could be a novel biomarker of glomerular damage.

FABP4 is thought to be a non-secretory protein since it lacks typical signal peptides [1], [2]. However, recent studies have shown that FABP4 is released from adipocytes [13], [14], [27] and that serum concentration of FABP4 is associated with obesity, insulin resistance, diabetes, hypertension, cardiac dysfunction, atherosclerosis and inflammatory markers [14]–[20], [28]. We and others showed that elevation of serum FABP4 was a novel predictor of cardiovascular prognosis [29]–[31]. Furthermore, a recent study revealed that FABP4 secreted from adipocytes controlled hepatic glucose production, leading to insulin resistance as an adipokine both *in vivo* and *in vitro* [13]. In addition, there is the possibility that circulating FABP4 has untoward effects on the vascular endothelium since a recent study showed that exogenous FABP4 inhibited endothelial nitric oxide synthase (eNOS) expression and its activation in human umbilical vascular endothelial cells [32]. In the present study, U-FABP4 was weakly correlated with S-FABP4 but not with BMI or waist circumference, while S-FABP was significantly correlated with BMI and waist circumference, suggesting that main source of U-FABP4 is derived from ectopic expression of glomerular FABP4 rather than increased adiposity and that locally increased FABP4 in the glomerulus affects renal dysfunction.

It has been reported that FABP4 expression is not detected in podocytes [21], [23]. An understanding of the role of podocytes as a glomerular filtration barrier has been advanced in the past decade [33]. However, the importance of glomerular endothelial cells in the pathogenesis of proteinuria has received attention recently [34], [35]. Loss of the glycocalyx in glomerular endothelial cells was shown to promote passage of albumin across the glomerular filtration barrier [34], [36]. Glomerular endothelial cell damage preceded podocyte injury in different types of renal injuries and decreased eNOS [35], [37], [38]. Interestingly, a study [39] showed that FABP4 expression decreased phosphorylation of eNOS and NO production in microvascular endothelial cells, contributing to endothelial dysfunction. Thus, there is the possibility that glomerular FABP4, which is up-regulated by endothelial damage, compromises NO production, leading to a vicious cycle of glomerular injury and increase in protein permeability.

It has been reported that U-FABP1 reflects damage of proximal tubular epithelial cells [4], [5] and predicts progression of renal dysfunction [6], [7]. Significance of U-FABP1 and U-FABP4 would be different, since it has been suggested that U-FABP4 reflects damage of glomerular damage [23]. In the present study, U-FABP4 was positively correlated with U-FABP1 (r = 0.534, p<0.001) (Table 3). Although UACR was positively correlated with U-FABP4 and U-FABP1 (Table 4), only U-FABP4, but not U-FABP1, was selected as an independent predictor of UACR in a stepwise regression analysis (Table 4), suggesting that U-FABP4 is potentially more sensitive predictor of albuminuria, especially in a population-based cohort.

There are some limitations of this study. First, it is not possible to critically address the causal relationship between U-FABP4 and renal dysfunction since we did not perform intervention for reducing glomerular FABP4. Second, U-FAPB4 was not measurable in nearly one-fourth of the subjects. Thus, the relationship between U-FAPB4 and albuminuria might not be generalized to the overall population. This issue needs to be re-examined if sensitivity of U-FABP4 assay is improved. In addition, some of subjects in the present study might have several drugs, including angiotensin II receptor blockers [40], [41] and statin [42], which have been reported to affect circulating FABP4 concentrations. Therefore, such drugs might modulate urinary excretion of FABP4. Lastly, since all study subjects were Japanese, whether the present findings can be generalized to other ethnicities remains unclear.

In conclusion, urinary FABP4 level is independently correlated with level of albuminuria and possibly predicts yearly decline of eGFR. U-FABP4 would be a novel biomarker of glomerular damage.
